# Impact of Urinary Incontinence on Quality of Life Among Women of Childbearing Age in Al Madinah Al Munawara, Saudi Arabia

**DOI:** 10.7759/cureus.24886

**Published:** 2022-05-10

**Authors:** Amna M Alshenqeti, Rawabi E Almutairi, Amal M Keram

**Affiliations:** 1 Family and Community Medicine, Prince Sultan Military Medical City, Riyadh, SAU; 2 Family Medicine, Family Medicine Academy, Al Madinah, SAU; 3 Family Medicine, Al Madinah Health Cluster, Al Madinah , SAU

**Keywords:** incontinence treatment, saudi women, urinary incontinence, kingdom of saudi arabia (ksa), quality of life (qol), urinary stress incontinence

## Abstract

Introduction

Urinary incontinence (UI) is a widely prevalent problem with a great impact on quality of life. It affects a person’s physical, social, occupational, and phycological aspects of life. Our study aimed to estimate prevalence, risk factors, effect on life, and help-seeking behaviors among women with urinary incontinence.

Methods

This is a cross-sectional analytic study conducted in primary health care centers among women of childbearing age in Al Madinah Al Munawara, Saudi Arabia. A total of 342 women aged between 18 to 50 years were included and interviewed using a previously validated and pretested Arabic version of the King's Health Questionnaire (KHQ).

Result

Out of 342 patients, 71 (20.8%) had UI. The mean age of the patients was 31.51 +9.36 years. Risk factors associated with UI were: BMI (p = 0.022, odds ratio = 1.06), multiparity (p = 0.027, odds ratio = 1.16), smoking (p = 0.018, odds ratio = 4.71), and chronic constipation (p = 0.013, odds ratio = 5.83). Only 28.2% of the affected women sought medical consultation. The main reasons for not seeking medical advice were the belief that UI is a common, normal aging process in 45%, while 14.1% were embarrassed by the condition, and 5.6% did not know that there was a treatment. Overall, there was a limitation in all domains of quality of life among patients who suffer from UI. The majority of limitations were slight to moderate. The most affected domain was sleep and energy.

Conclusion

UI is common and adversely affects the quality of life of women of childbearing age in Al Madinah Al Munawara. Obesity, multiparity, smoking, and chronic constipation are significant risk factors. Less than half of patients with UI sought medical care.

## Introduction

Urinary incontinence (UI) is a common condition, especially in women, with a profound impact on quality of life [1]. It is defined by the International Urogynecological Association (IUGA)/International Continence Society (ICS) as a complaint of involuntary pass of urine [2]. UI is classified into many types: (1) stress incontinence, which is an involuntary leak of urine on physical exertion or efforts such as coughing, sneezing, or laughing, (2) urgency incontinence in which the unintentional urine leak is synchronous with urgency, which defined as a sensation of a sudden, compelling desire to void that is difficult to defer, (3) mix incontinence, which is a combination of both stress and urgency urinary incontinence [2].

UI affects more than 423 million people around the world with an increased risk of three times in women than in men [3]. In North America, more than half of the women older than 45 suffer from UI [4]. However, it is often underreported by sufferers, because of misconception and consideration as a natural consequence of aging, giving birth, and due to a sense of shame [5]. The reported prevalence in the literature of UI worldwide ranges from 5% to 70% [1]. This wide variance is due to differences in case definition, population criteria, and sampling procedure [1]. In Saudi Arabia, the prevalence of UI was found to range from 29% to 56% [6-11].

Quality of life is defined as the degree to which a person enjoys important possibilities in his or her life. It reflects an individual's sense of well-being and satisfaction with life [12]. Although urinary incontinence is not considered a fatal disease, the loss of urine control has a significant effect on the quality of life [13,14]. It affects physical, occupational, social, psychological, and personal aspects of patients’ lives significantly [13,15]. The key consequences include loss of self-confidence and social isolation in addition to other negative outcomes such as anxiety, depression, and a decrease in physical activity [16]. Risk factors significantly attributed to increasing urinary incontinence include increased age, obesity, parity, cigarette smoking, diabetes mellitus, and hysterectomy [15].

This study aimed to determine the prevalence, risk factors, and impact of UI on quality of life among women of childbearing age in Al Madinah Al Munawara, Saudi Arabia.

## Materials and methods

This study is a multicenter cross-sectional analytic study done in 10 primary health care centers (PHCCs) in Al Madinah Al Munawara. The study was approved by the Institutional Review Board, General Directorate of Health Affairs in Al Madinah (approval number H-03-M-084). A representative sample of 342 participants was calculated using Epi Info™/OpenEpi (Centers for Disease Control and Prevention (CDC), Atlanta, Georgia, United States) free statistical software at confidence interval 95%, and 5% margin of error. Women of childbearing age who visit PHCCs were chosen randomly and invited to participate. Inclusion criteria were any women of childbearing age from 18 to 50 years, with or without comorbidities, who agreed to participate. Those who had delivered in the last three months, had gynecological or lower urinary tract surgery during the previous three months, and post-menopause women were excluded from the study. Informed consent was obtained prior to administering the questionnaire through a face-to-face interview. Definition of UI and its types was adopted from International Urogynecological Association (IUGA)/International Continence Society (ICS) as any leakage or involuntary loss of urine [2]. For the purpose of the study, the definition was confined to an incidence during last year. The final questionnaire contained sections on the sociodemographic characteristics, risk factors for UI, daily habits, gynecological information, experience of UI, severity of UI, and medical-seeking behavior for UI.

The impact of urinary incontinence on quality of life was assessed using a previously validated and pretested Arabic version of The King's Health Questionnaire (KHQ) [17]. KHQ is a disease-specific health-related quality of life (HRQOL) instrument for measuring QOL in women with UI. KHQ is widely used and proven to be valid and reliable [12]. In comparison to other tools used to measure the quality of life in women with UI, KHQ is the simplest to administer, easily understandable by the participant, and covers several domains of life [12]. KHQ has three parts consisting of 21 items. Part 1 contains general health perception and incontinence impact (one item each). Part 2 contains role limitations, physical limitations, social limitations (two items each), personal relationships, emotions (three items each) and sleep/energy (two items), severity measures (four items). Part 3 is considered a single item and contains 10 responses in relation to frequency, nocturia, urgency, urge, stress, intercourse incontinence, nocturnal enuresis, infections, pain, and difficulty in voiding. The responses in KHQ have a four-point rating system. The eight subscales (“domains”) score between 0 (best) and 100 (worst). The Symptom Severity scale is scored from 0 (best) to 30 (worst). Note that the lower scores indicate patient well-being and higher scores mean that the person is suffering and severely affected by UI [12].

Data analysis was performed using Statistical Package for the IBM SPSS Statistics for Windows, Version 23.0 (Released 2015; IBM Corp., Armonk, New York, United States). Frequency and percentages were used to display categorical variables. Minimum, maximum, mean, and standard deviation were used to present numerical variables. The Chi-square test was used to test for the presence of an association between categorical variables. Multivariate logistic regression was used to determine the risk factors for having a UI among women of childbearing age. The logistic regression model included the following variables: age, BMI, current pregnancy status, parity, history of gynecological surgeries, smoking history, diabetes, hypertension, asthma, and chronic constipation. The level of significance was set at 0.05.

## Results

A total of 342 patients were included in the study. Table 1 shows the sociodemographic profile of the patients. The mean age of patients was 31.51 +9.36. As for marital status, 93 (27.2%) were single, 228 (66.7%) were married, 16 (4.7%) were divorced, and five (1.5%) were widowed. BMI wise, 20 (5.8%) were underweight, 111 (32.5%) had a normal weight, 103 (30.1%) were overweight, 104 (30.4%) were obese, and four (1.2%) did not have a documented BMI. 

**Table 1:** Sociodemographic profile of the patients (n = 342)

Demographical Characteristics		n	%
Age			
Mean		31.51	
Standard deviation		9.36	
Marital status			
Single		93	27.20
Married		228	66.70
Divorced		16	4.70
Widowed		5	1.50
Nationality			
Saudi		317	92.70
Non-Saudi		25	7.30
Education			
Illiterate		9	2.60
Write and read		2	0.60
Primary school		11	3.20
Intermediate school		18	5.30
High school		104	30.40
Bachelor's degree		184	53.80
Higher education (Masters/PhD)		14	4.10
Job			
Employee		81	23.70
Unemployed		261	76.30
BMI class			
Underweight		20	5.80
Normal weight		111	32.50
Overweight		103	30.10
Obese		104	30.40
Undocumented		4	1.20

Table 2 displays the surgical and gynecological profile and association with UI. As for the gynecological profile, 66 (19.3%) reported being pregnant during the study, while 276 (80.7%) were not. Current pregnancy status was not associated with an increased risk of UI (P= 0.661). The mean number of times the patients had been pregnant was 2.58 +2.74. The mean number of times the patients had undergone vaginal delivery was 1.71 +2.31. As for the number of cesarean sections the participants had undergone, 264 (77.2%) did not undergo cesarean births at all, 37 (10.8%) had undergone cesarean section once, 15 (4.4%) had undergone it twice, 17 (5%) have undergone it thrice, five (1.5%) have undergone it four times, and four (1.2%) have undergone it five times. History of cesarean section was found to be significantly associated with UI (P=0.031). Most of the participants had never undergone vacuum/forceps-assisted vaginal delivery 319 (93.3%), while 23 (6.7%) have undergone it once, which was found to be associated with UI (P=0.025). Of the participants, 254 (74.3%) had no history of abortion at all, while 56 (16.4%) had undergone abortion once, 24 (7%) had undergone it twice, five (1.5%) had undergone it thrice, two (0.6%) had undergone it four times, and one (0.3%) had undergone it five times. History of abortion was found to have a significant association with UI. As for the surgical profile of the patients, 30 (8.8%) had undergone abdomen gynecologic surgery, and 22 (6.4%) had undergone vaginal gynecologic surgery, P<0.001 and P=0.814, respectively. 

**Table 2:** Gynecological and surgical profile of the patients (n = 342) * Significant at level 0.05

Question	n	%	Urinary incontinence		P-Value
			Yes n (%)	No n (%)	
Gynecological Profile					
Current pregnancy					
Yes	66	19.3	15 (22.7%)	51 (77.3%)	0.661
No	276	80.7	56 (20.3%)	220 (79.7%)	
History of cesarean births					
Yes	78	22.8	23 (29.5%)	55 (70.5%)	0.031*
No	264	77.2	48 (18.2%)	216 (81.8%)	
History of vacuum/forceps assisted vaginal deliveries					
Yes	23	6.7	9 (39.1%)	14 (60.9%)	0.025*
No	319	93.3	62 (19.4%)	257 (80.6%)	
History of abortions					
Yes	88	25.7	29 (33.0%)	59 (67.0%)	0.001*
No	254	74.3	42 (16.5%)	212 (83.5%)	
Number of pregnancies					
Mean standard deviation	2.58 + 2.74				
Number of normal vaginal deliveries					
Mean standard deviation	1.71 + 2.31				
Surgical Profile					
History of abdomen gynecologic surgery					
Yes	30	8.8	14 (46.7%)	16 (53.3%)	<0.001*
No	312	91.2	57 (18.3%)	255 (81.7%)	
History of vaginal gynecologic surgery					
Yes	22	6.4	5 (22.7%)	17 (77.3%)	0.814
No	320	93.6	66 (20.6%)	254 (79.4%)	

**Figure 1:**
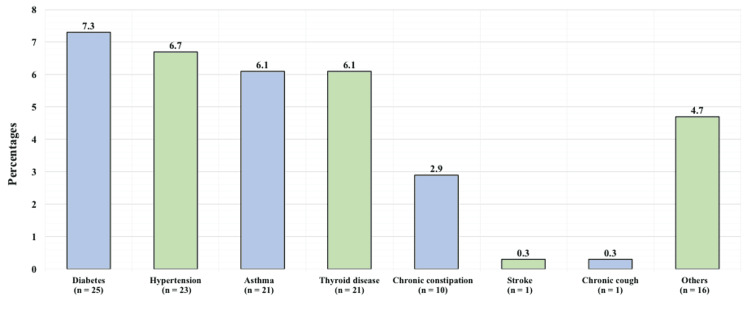
Patients' co-morbidities

According to the medical history of the patients, 95 (27.8%) reported having a chronic disease, 131 (38.3%) reported exercising regularly (at least three times a week for at least 20 minutes), and 13 (3.8%) were smokers. Figure 1 demonstrates the patients' co-morbidities. The most commonly reported co-morbidities were diabetes in 25 (7.3%), hypertension in 23 (6.7%), asthma in 21 (6.1%), and thyroid disease in 21 (6.1%). 

The prevalence of UI was 20.8% (71 patients). Table 3 shows the profile of UI. As for how often the patients experience UI, 41 (57.7%) reported once weekly, 14 (19.7%) reported two to three times a week, six (8.5%) reported once daily, four (5.6%) reported more than once a day, and six (8.5%) reported all the time. As for the leaked amount of urine, 54 (76%) reported it was just a minimal amount, 16 (22.5%) reported it was a moderate amount, and one (1.4%) reported it was a high amount. A total of 25 (35.2%) patients reported having disclosed to someone, while 46 (64.8%) never did. Only 20 (28.2%) reported seeking medical consultation, while 51 (71.8%) did not. For those who sought medical attention, two (10%) reported that the problem was not solved, seven (35%) reported the problem was partially solved, and 11 (55%) reported that the problem was completely solved. As for the patients rating out of 10 on how much the urinary incontinence affected their daily life, the mean was 3.48 +3.04.

**Table 3:** Profile of urinary incontinence (n = 71)

Question	n	%
How often do you experience urinary incontinence?		
Once weekly	41	57.7
Two to three times a week	14	19.7
Once daily	6	8.5
More than once a day	4	5.6
All the time	6	8.5
How much would you estimate the amount of the leaked urine?		
Minimal amount	54	76
Moderate amount	16	22.5
High amount	1	1.4
Have you disclosed to someone close to you about this problem?		
Yes	25	35.2
No	46	64.8
Have you sought a medical consultation for your urinary incontinence problem?		
Yes	20	28.20
No	51	71.80
What was the result of the medical consultation you sought? (n = 20)		
Problem not solved	2	10.00
Problem partially solved	7	35.00
Problem completely solved	11	55.00
Out of 10 how would you rate the effect of urinary incontinence on your daily life		
Minimum	0	
Maximum	10	
Mean	3.48	
Standard deviation	3.04	

**Figure 2:**
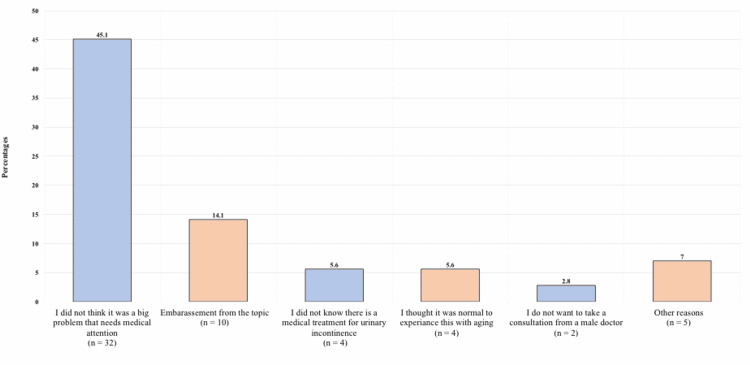
Barriers for seeking medical care for urinary incontinence

Figure 2 displays the barriers for which patients with UI didn’t seek medical attention. The most commonly reported reason was not thinking it was a big problem that needs medical care in 32 patients (45.1%), embarrassment from the condition in 10 (14.1%), and not knowing that there was a treatment for urinary incontinence in four (5.6%). 

Table 4 and Table 5 present the quality of life assessment of patients with UI using the KHQ. Table 4 displays part 1: general health perception and incontinence impact, and part 2: role limitations, physical limitations, social limitations, personal relationships, emotions, sleep/energy, and severity measures. Table 5 displays part 3 of the KHQ: symptom severity scale. 

**Table 4:** Quality of life assessment for patients with urinary incontinence using King's Health Questionnaire (Part 1 and 2) (n = 71)

Question	n	%
Part 1: General Health Perception and Incontinence Impact		
How would you describe your health at the present?		
Very good	37	52.1
Good	24	33.8
Fair	9	12.7
Poor	1	1.4
How much do you think your bladder problem affects your life?		
Not at all	28	39.4
A little	26	36.6
Moderately	10	14.1
A lot	7	9.9
Part 2: Role Limitations		
Does your bladder problem affect your household tasks? (Cleaning, shopping etc)		
Not at all	47	66.2
Slightly	20	28.2
Moderately	4	5.6
A lot		
Does your bladder problem affect your job or your normal daily activities outside the home?		
Not at all	39	54.90
Slightly	18	25.40
Moderately	14	19.70
A lot		
Part 2: Physical Limitations		
Does your bladder problem affect your physical activities (e.g. going for a walk, running, sport, gym etc)?		
Not at all	44	62.00
Slightly	21	29.60
Moderately	4	5.60
A lot	2	2.80
Does your bladder problem affect your ability to travel?		
Not at all	56	78.90
Slightly	8	11.30
Moderately	5	7.00
A lot	2	2.80
Part 2: Social Limitations		
Does your bladder problem limit your social life?		
Not at all	51	71.80
Slightly	13	18.30
Moderately	7	9.90
A lot		
Does your bladder problem limit your ability to see and visit friends?		
Not at all	57	80.30
Slightly	11	15.50
Moderately	3	4.20
A lot		
Part 2: Personal Relationships		
Does your bladder problem affect your relationship with your partner?		
Not applicable	43	60.60
Not at all	27	38.00
Slightly	1	1.40
Moderately	0	
A lot	0	
Does your bladder problem affect your sex life?		
Not applicable	42	59.20
Not at all	21	29.60
Slightly	7	9.90
Moderately	1	1.40
A lot	0	
Does your bladder problem affect your family life?		
Not applicable	45	63.40
Not at all	24	33.80
Slightly	2	2.80
Moderately	0	
A lot	0	
Part 2: Emotions		
Does your bladder problem make you feel depressed?		
Not at all	52	73.20
Slightly	13	18.30
Moderately	5	7.00
Very much	1	1.40
Does your bladder problem make you feel anxious or nervous?		
Not at all	39	54.90
Slightly	17	23.90
Moderately	10	14.10
Very much	5	7.00
Does your bladder problem make you feel bad about yourself?		
Not at all	46	64.80
Slightly	15	21.10
Moderately	8	11.30
Very much	2	2.80
Part 2: Sleep / Energy		
Does your bladder problem affect your sleep?		
Never	35	49.30
Sometimes	23	32.40
Often	8	11.30
All the time	5	7.00
Does your bladder problem make you feel worn out and tired?		
Never	45	63.40
Sometimes	19	26.80
Often	4	5.60
All the time	3	4.20
Part 2: Severity measures		
Wear pads to keep dry?		
Never	33	46.50
Sometimes	19	26.80
Often	12	16.90
All the time	7	9.90
Be careful how much fluid you drink?		
Never	26	36.60
Sometimes	16	22.50
Often	20	28.20
All the time	9	12.70
Change your underclothes because they get wet?		
Never	24	33.80
Sometimes	23	32.40
Often	12	16.90
All the time	12	16.90
Worry in case you smell?		
Never	37	52.10
Sometimes	13	18.30
Often	12	16.90
All the time	9	12.70

**Table 5:** Quality of life assessment of patients with urinary incontinence using King's Health Questionnaire (Part 3) (n = 71)

Symptom	Never	A little	Moderately	A lot
Part 3: Symptom Severity Scale				
Frequency: going to the toilet very often	16 (22.5%)	16 (22.5%)	25 (35.2%)	14 (19.7%)
Nocturia: getting up at night to pass urine	0 (0%)	39 (54.9%)	20 (28.2%)	12 (16.9%)
Urgency: a strong and difficult to control desire to pass urine	0 (0%)	42 (59.2%)	18 (25.4%)	11 (15.5%)
Urge incontinence: urinary leakage associated with a strong desire to pass urine	0 (0%)	44 (62%)	15 (21.1%)	12 (16.9%)
Stress incontinence: urinary leakage with physical activity (e.g., coughing, running)	0 (0%)	37 (10.8%)	18 (5.3%)	16 (4.7%)
Nocturnal enuresis: wetting the bed at night	0 (0%)	69 (97.2%)	2 (0.6%)	0 (0%)
Intercourse incontinence: urinary leakage with sexual intercourse	0 (0%)	68 (95.8%)	3 (4.2%)	0 (0%)
Bladder pain	0 (0%)	58 (81.7%)	9 (12.7%)	4 (5.6%)
Infections	60 (84.5%)	8 (11.3%)	2 (2.8%)	1 (1.4%)

Table 6 displays the scores calculated for each domain and each part of KHQ. The mean score of part 1 (general health perception and incontinence impact) was 47.3 + 37.02. For part 2 (role limitations, physical limitations, social limitations, personal relationships, emotions, sleep/energy, severity measures), the mean was 130.28 + 106.98. For part 3 (symptom severity scale), the mean was 11.27 + 2.93. 

**Table 6:** Quality of life assessment of patients with urinary incontinence using King's Health Questionnaire (n = 71)

Item	Minimum	Maximum	Mean	Standard deviation
Domain 1: General health perception	0	75	15.85	19.01
Domain 2: Incontinence impact	0	100	31.46	32.31
Domain 3: Role limitations	0	67	17.37	20.79
Domain 4: Physical limitations	0	83	13.85	19.52
Domain 5: Social limitations	0	67	9.00	16.49
Domain 6: Personal relationships	0	133	16.43	25.12
Domain 7: Emotions	0	78	18.00	22.25
Domain 8: Sleep / energy	0	100	21.13	23.56
Domain 9: Severity measures	0	100	34.51	28.43
Part 1: General Health Perception and Incontinence Impact.	0	150	47.30	37.02
Part 2: Role limitations, Physical limitations, social limitations, Personal relationships, Emotions, Sleep/Energy, Severity measures	0	528	130.28	106.98
Part 3: Symptom Severity Scale	7	18	11.27	2.93

Table 7 demonstrates the factors associated with the presence of UI. Older age, higher BMI, parity, history of abdominal or vaginal gynecological surgery, diabetes mellitus, and hypertension were significantly associated with the presence of UI (p = 0.001). Chronic constipation and smoking were also significantly associated with UI (p = 0.021) for both. On the other hand, bronchial asthma and being currently pregnant were both not significantly associated with the presence of UI among women of childbearing age.

**Table 7:** Factors associated with the presence of urinary incontinence

Factor	Presence of Urinary Incontinence			P-Value		
	No	Yes				
Are you currently pregnant? (n, %)			0.661			
Yes	51 (77.3%)	15 (22.7%)				
No	220 (79.7%)	56 (20.3%)				
History of abdominal or vaginal gynecological surgery (n, %)			0.001*			
Yes	29 (61.7%)	18 (38.3%)				
No	242 (82%)	53 (18%)				
Having diabetes mellitus (n, %)			< 0.0001*			
Yes	13 (52%)	12 (48%)				
No	258 (81.4%)	59 (18.6%)				
Having Hypertension (n, %)			0.001*			
Yes	12 (52.2%)	11 (47.8%)				
No	259 (81.2%)	60 (18.8%)				
Having asthma (n, %)			0.362			
Yes	15 (71.4%)	6 (28.6%)				
No	256 (79.8%)	65 (20.2%)				
Having chronic constipation (n, %)			0.021*			
Yes	5 (50%)	5 (50%)				
No	266 (80.1%)	66 (19.9%)				
Are you a smoker? (n, %)			0.021*			
Yes	7 (53.8%)	6 (46.2%)				
No	264 (80.2%)	65 (19.8%)				
Age (mean, standard deviation)	30.13 + 8.86	36.79 + 9.42	< 0.001*			
BMI (mean, standard deviation)	26.69 + 5.97	30.57 + 8.06	< 0.001*			
Number of parity (mean, standard deviation)	2.17 + 2.4	4.17 + 3.35	< 0.001*			
*Significant at level 0.05						

Table 8 illustrates the multivariate logistic regression (factors predicting the presence of UI among women of childbearing age). The logistic regression model included the following factors: age, BMI, current pregnancy status, number of pregnancies, history of gynecological surgeries, history of smoking, diabetes, hypertension, asthma, and chronic constipation. The following factors predicted a significantly higher risk for having UI: BMI (p = 0.022, odds ratio = 1.06), number of pregnancies (p = 0.027, odds ratio = 1.16), being a smoker (p = 0.018, odds ratio = 4.71), and having chronic constipation (p = 0.013, odds ratio = 5.83).

**Table 8:** Multivariate logistic regression (factors predicting the presence of urinary incontinence among women of childbearing age) * Significant at level 0.05

Factor		P-Value	Odds Ratio	Confidence Interval	
Age		0.263	1.03	0.98	1.07
BMI		0.022*	1.06	1.01	1.11
Currently pregnancy (yes vs no)		0.124	1.75	0.86	3.57
Number of pregnancies		0.027*	1.16	1.02	1.32
History of previous abdominal or vaginal gynecological surgery (yes vs no)		0.186	1.67	0.78	3.57
Diabetes mellitus (yes vs no)		0.063	2.67	0.95	7.50
Smoking (yes vs no)		0.018*	4.71	1.30	17.02
Hypertension (yes vs no)		0.698	1.24	0.42	3.72
Bronchial asthma (yes vs no)		0.528	1.46	0.45	4.75
Chronic constipation (yes vs no)		0.013*	5.83	1.45	23.36

## Discussion

UI is a global medical problem that is more prevalent in females [1]. The prevalence of UI among women in our study was 20.8% (71 patients), which lies within the worldwide prevalence range of 5-70% [1]. This figure is similar but slightly lower than the previous studies done in Saudi Arabia, which reported a prevalence between 29-56% in Riyadh [6,11], 34.3-41.4% in Jeddah [7,8], and 47.5% in Asir [10]. Other middle-eastern countries reported a concordance prevalence of 20.7% among Qatari women [18]. Higher percentages were reported in United Arab Emirates, Kuwait, and Egypt at 42.2%, 54.5%, and 54.8%, respectively [19,20,21]. Internationally, in the United States, 25-50% of women suffer from UI [22]. This is similar to the reported prevalence in Canada (51%) [23]. This variation in the prevalence can be attributed to the differences in study populations selected based on different inclusion and exclusion criteria and the sample size in those studies. Furthermore, the different definitions of UI that have been used and how each study have differently identified participants with UI might have influenced the estimation of UI prevalence across different studies.

Knowing and identifying risk factors for UI is the mainstay of management. The present study identified several risk factors and associated factors for UI. Obesity, multiparity, smoking, and chronic constipation were found to be significant risk factors for urinary incontinence in the present study; BMI (p = 0.022, odds ratio = 1.06), parity (p = 0.027, odds ratio = 1.16), smoking (p = 0.018, odds ratio = 4.71), and chronic constipation (p = 0.013, odds ratio = 5.83). Furthermore, diabetes mellitus, hypertension, older age, vaginal surgeries, device-assisted birth, and history of abortion were found to be significantly associated with UI. This is similar to Altaweel et al.'s findings of older age, obesity, vaginal surgery, high parity, and diabetes mellitus as risk factors for UI among women in Riyadh [6]. Similarly, Al-Bader et al. in Jeddah identified older age (postmenopausal), greater parity, chronic cough, constipation, and diabetes mellitus as significant risk factors for urinary incontinence [8]. In parallel, El-Azab et al. in Egypt reported that menopause, higher parity (>3), vaginal delivery, and previous multiple abortions (>3) were significantly associated with UI [21]. On the other hand, bronchial asthma was found to be significantly related to UI in Qatar [18], which is contrary to our study results (P = 0.362). 

Almost two-thirds of women who had UI did not consider leakage as a problem and 60% of women who suffer from urine loss had never seek medical help for it [23]. The present study found that 64.8% of patients never disclose the problem to someone. Only 28.2% reported seeking medical consultation, while 71.8% never did. This low percentage is similar to previous results done in the same region, (14.5%) in Jeddah, and (16.3%) in Riyadh [8,17]. Of the women in this study, 45% did not find UI to be a big enough problem needing medical attention, 14.1% were embarrassed from the condition, and 5.6% did not know that there was a treatment. Likewise, in a systematic review done by Hammad et al. about UI in the Gulf countries, they reported that the main reasons for not seeking medical advice were an embarrassment to see doctors, especially male doctors, and the belief that UI is common, not abnormal, or untreatable [24]. 

In our study, overall, there was a limitation in all domains of quality of life among patients who suffer from UI. The majority of limitations were slight to moderate. These results are consistent with previous studies [6,8,17,24]. Altaweel and Alharbi in their work reported that UI did not impact patients significantly (less than 10% in each and all five areas and life: housework, attending entertainment events, physical activities, traveling by car more than 30 min from home, and social gatherings [6]. The most affected domain in the present study was sleep and energy. As half of the affected women reported that UI had affect their sleep badly on different degrees from mild to severe (Mean 21.13 + 23.56). This is similar to the finding reported by Bakarman et al. as they reported sleep and energy were the most affected in which the percentage of limitations reached 27.1% [7]. Considering the impact on emotions, 38% of patients in the present study admitted that they, slightly or moderately, feel anxious or nervous, while 26% admitted to feeling depressed. Similarly, Mallah et al. in Iran reported a significant impact on the quality of life generally and mental health specifically among women with UI compared to women without UI (P = 0.002 and 0.017, respectively) [25]. 

This is the first study conducted in Al Madinah Al Munawara region on the topic. It highlighted the prevalence, risk factors, severity, and the effect of the UI on the daily life of women. A potential limitation of this study is that the diagnosis of UI was based on subjective complaints by patients and wasn’t confirmed by clinical examination. Further studies in older population should be carried out to investigate the problem in that age. 

## Conclusions

Urinary incontinence is common and adversely affects the quality of life of women of childbearing age in Al Madinah Al Munawara. Obesity, multiparity, smoking and chronic constipation are significant risk factors for UI. Less than half of the affected patients with UI sought medical care due to misconception of the problem as a normal or expected process and embarrassment from discussing it. Some of the risk factors for UI are modifiable, which should attract attention to be corrected. We recommend further health campaigns to enhance the awareness of women about UI and encourage them to seek medical help.
